# Uremic Toxin Lanthionine Induces Endothelial Cell Mineralization In Vitro

**DOI:** 10.3390/biomedicines10020444

**Published:** 2022-02-14

**Authors:** Annapaola Coppola, Carmela Vigorito, Patrizia Lombari, Yuselys García Martínez, Margherita Borriello, Francesco Trepiccione, Diego Ingrosso, Alessandra F. Perna

**Affiliations:** 1 Department of Precision Medicine, University of Campania “Luigi Vanvitelli”, Via L. De Crecchio 7, 80138 Naples, Italy; annapaola.coppola@unicampania.it (A.C.); patrizia.lombari@unicampania.it (P.L.); margherita.borriello@unicampania.it (M.B.); 2 Department of Translational Medical Science University of Campania “Luigi Vanvitelli”, Via Pansini, Bldg 17, 80131 Naples, Italy; carmela.vigorito@unicampania.it (C.V.); yuselys.garciamartinez@unicampania.it (Y.G.M.); francesco.trepiccione@unicampania.it (F.T.)

**Keywords:** endothelial cells, uremic toxins, lanthionine, calcium/phosphate dysmetabolism, uremia, chronic kidney disease

## Abstract

Vascular calcification (VC) is a pathological event caused by the unusual deposition of minerals in the vascular system, representing the leading cause of cardiovascular mortality in chronic kidney disease (CKD). In CKD, the deregulation of calcium and phosphate metabolism, along with the effect of several uremic toxins, act as key processes conveying altered mineralization. In this work, we tested the ability of lanthionine, a novel uremic toxin, to promote calcification in human endothelial cell cultures (Ea.hy926). We evaluated the effects of lanthionine, at a concentration similar to that actually detected in CKD patients, alone and under pro-calcifying culture conditions using calcium and phosphate. In pro-calcific culture conditions, lanthionine increased both the intracellular and extracellular calcium content and induced the expression of Bone Morphogenetic Protein 2 (*BMP2*) and RUNX Family Transcription Factor 2 (*RUNX2*). Lanthionine treatment, in pro-calcifying conditions, raised levels of tissue-nonspecific alkaline phosphatase (*ALPL*), whose expression also overlapped with Dickkopf WNT Signaling Pathway Inhibitor 1 (*DKK1*) gene expression, suggesting a possible role of the latter gene in the activation of ALPL. In addition, treatment with lanthionine alone or in combination with calcium and phosphate reduced Inorganic Pyrophosphate Transport Regulator (*ANKH*) gene expression, a protective factor toward the mineralizing process. Moreover, lanthionine in a pro-calcifying condition induced the activation of ERK1/2, which is not associated with an increase in DKK1 protein levels. Our data underscored a link between mineral disease and the alterations of sulfur amino acid metabolisms at a cell and molecular level. These results set the basis for the understanding of the link between uremic toxins and mineral-bone disorder during CKD progression.

## 1. Introduction

Vascular calcification (VC) is a pathological process that may occur in patients with chronic kidney disease (CKD). Individuals affected by CKD have an elevated cardiovascular risk, where the risk of calcification increases with disease progression [1]. No specific biomarkers have been identified to adequately monitor the risk of developing VC. However, VC is often associated with various factors, such as old age, diabetes and alterations of mineral metabolism, with special regard to the deregulation of calcium and phosphate metabolism [2]. The deposition of calcium-phosphate salts in blood vessels is closely linked to elevation of serum phosphate levels and to transient hypercalcemia [3]. Phosphate is present in all fluids and plays a key role in various biological processes, such as energy metabolism, cell signaling and the stabilization of phospholipids on cell membranes [4]. Intracellular phosphate concentration is influenced by pH, hormones and cellular localization, where phosphate homeostasis is maintained by cellular and endocrine sensors [5]. In the kidney, phosphate reabsorption depends on parathyroid hormone (PTH), fibroblast growth factor 23 (FGF23) and dietary phosphate, but in CKD patients, hyperphosphatemia is a complication independent of PTH and calcitriol levels [6,7,8]. In addition to high phosphate levels, altered calcium (Ca^2+^) homeostasis is another factor affecting mineralization [9]. Ca^2+^ modulates the function of the parathyroid gland, the thyroid gland, the kidneys and other organs via the calcium-sensing receptors (CaSR) [10]. Decreased plasma calcium inactivates CaSR, leading to an increase in PTH secretion with direct effects on renal reabsorption and bone resorption. An increase in PTH levels promotes intestinal calcium absorption, which, in turn, is regulated by 1,25-dihydroxy vitamin-D [11]. 1,25-dihydroxy vitamin-D is the active metabolite of vitamin D, able to induce specific gene transcription once bound to the vitamin D receptor. One of the most important functions performed by vitamin D is the maintenance of serum Ca^2+^ and phosphate homeostasis [12]. The final step of 1,25-dihydroxy vitamin-D synthesis, where 25-hydroxy vitamin D is hydroxylated to 1,25-(OH)_2_D, is catalyzed by kidney 1-α hydroxylase. However, in CKD, this enzyme displays a lower activity, resulting in a 1,25-dihydroxyvitamin D reduction [13,14]. In these subjects, because of the loss of renal mass and the increase in phosphate retention, 1,25-dihydroxyvitamin D levels start to decline in the early stages of kidney disease, promoting the expression of FGF23 [15]. FGF23 is a bone-derived hormone that promotes phosphaturia and reduces the synthesis of 1,25-dihydroxyvitamin D by inhibiting 1-α hydroxylase activity [16]. In normal conditions, the stimuli that promote FGF23 expression are the high level of 1,25-dihydroxyvitamin D and the increase in phosphate dietary intake. Conversely, in the long term, with the functional and structural renal decline found in CKD subjects, this mechanism becomes a dysfunctional process. This consists of a prominent reduction of 1,25-dihydroxyvitamin D associated with PTH overproduction, resulting in secondary hyperparathyroidism [2,17].

**Figure 1:**
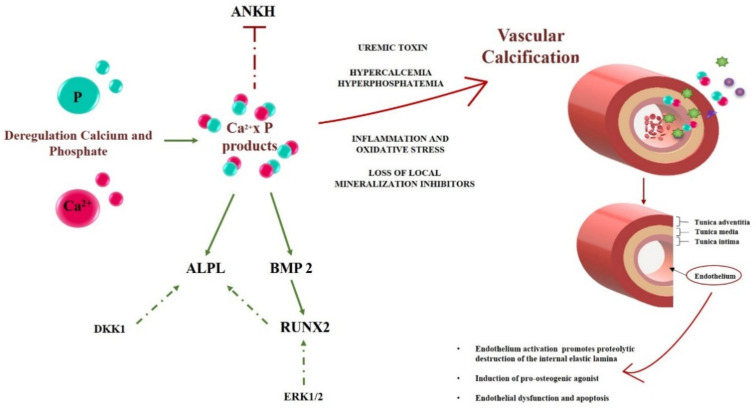
A schematic representation of the early steps of the calcification process in CKD. Deregulation of calcium (Ca^2+^, in red) and phosphate (P, in green) are key events initiating and promoting vascular calcification. Various genes and proteins involved in the endothelium mineralization are reported in the left part of the figure, where active and inactive genes are underscored in a green or red color, respectively. The dashed green lines represent a secondary pathway involved in gene activation. With various contributions from uremic toxins, inflammation and oxidative stress, as well as the loss of local inhibitors, the system evolves toward calcification, according to mechanisms which are still unclear and partly related to the scope of the present work. On the right half of the Figure, a schematic structure of a blood vessel and the role of the endothelium modifications are reported. Calcium, Ca^2+^; Phosphate, P; Inorganic Pyrophosphate Transport Regulator, *ANKH*; Bone Morphogenetic Protein 2, *BMP2*; tissue-nonspecific alkaline phosphatase, *ALPL*; RUNX Family Transcription Factor 2, *RUNX2*; Dickkopf WNT Signaling Pathway Inhibitor 1, *DKK1*; Extracellular signal-regulated 1/2, ERK1/2.

Three layers characterize the vascular wall: intima, media and adventitia. The intima consists of endothelium and sub-endothelial connective tissue, separated from the media by the internal elastic lamina [18]. Various studies show that endothelial cells (ECs) are involved in the regulation of mineralization [19,20,21,22]. In fact, the endothelium represents the main constituent of blood vessels, in direct contact with blood flow, and is influenced by various serum stimuli, such as inflammatory factors, vasoactive agents and phosphate, that promote endothelial dysfunction and facilitate arterial calcification [23]. These processes may be further favored by the accumulation of uremic toxins in CKD [24]. Specific genes and proteins are involved in the mineralization process at the molecular level. One initial harmful effect on the endothelium, during the vessel wall mineralization process, is the disruption of the internal elastic lamina. This is a flexible barrier between endothelial cells and smooth muscular cells (SMCs), allowing for the passage of various vasoactive factors. Abnormalities in this site alter the normal equilibrium between the intima and media, allowing the calcification agonists to migrate from the endothelium to the other layers [25]. An example of an agonist is Bone Morphogenetic Protein 2 (*BMP2*), which is sensitive to the presence of calcium-phosphate products (CaxP), which could promote the pro-calcific shift in neighboring cells [26,27,28]. The complex dynamics of phosphate/pyrophosphate (Pi/PPi) homeostasis are regulated by tissue-nonspecific alkaline phosphatase (*ALPL*) and by Inorganic Pyrophosphate Transport Regulator (*ANKH*). *ALPL* boosts the osteogenic effect of the endothelium, bringing the extracellular accumulation of phosphate, which derives from the hydrolysis of pyrophosphate [28]. Instead, *ANKH* plays a protective role, inhibiting hydroxyapatite formation and calcium deposition [29]. High serum phosphate in CKD, together with uremic toxins, can induce the expression of *ALPL* with the increase of phosphate as a powerful stimulator of endothelium damage [30]. However, the role of *ALPL* on the endothelium is not yet fully understood, although studies demonstrated that *ALPL* expression preceded the initial calcium deposition and the expression of other agonists such as RUNX Family Transcription Factor 2 (*RUNX2*) and *BMP2* [30]. Various studies demonstrated that the expression of *ALPL* was promoted by Dickkopf WNT Signaling Pathway Inhibitor 1(*DKK1*) [3,30] and *RUNX2* [31]. An increased expression of the latter genes, together with the activation of various molecular signals, create the perfect setting to promote the mineralization process. The activation of extracellular signal-regulated kinases 1/2 (ERK1/2) is promoted by high phosphate and, in turn, induces *RUNX2* phosphorylation, increasing its transcriptional activity [32,33]. A schematic representation of this molecular mechanism and some crucial genes and proteins involved in the earliest phase of the mineralizing process are reported in Figure 1. Concerning uremic toxins, in our previous work, we demonstrated that, in CKD patients, lanthionine, a side product of sulfur amino acid metabolism, was able to alter calcium homeostasis, and was also associated with inflammation, which were related to each other [34]. Now we attempt to prove if lanthionine could be able to alter the expression of specific markers in endothelial cell cultures that are involved in the initial stages of the mineralization process.

## 2. Materials and Methods

### 2.1. Cell Culture

Human umbilical vein cells, EA.hy926 (ATCC), were cultured in DMEM medium with 10% Fetal Bovine Serum (S1810-500, Microtech, Pozzuoli, NA, Italy), 2 mM L-glutamine (X0550-100, Microtech, Pozzuoli, NA, Italy) and 0.1% penicillin–streptomycin (L0022-100, Microtech, Pozzuoli, NA, Italy). Cells were grown at 37 °C in a humidified atmosphere with 5% CO_2_.

### 2.2. Experimental Design

The EA.hy926 cells were treated the day after they were seeded. The cells were treated with 1 μM DL-lanthionine (Lan, L0010, TCI, Tokyo, Japan) for 24 h, 48 h, 96 h and 10 days. DL-lanthionine was dissolved in 1 M HCl in water at a 50 mg/mL (240 mM) concentration. The effect of the vehicle, in terms of HCl addition to DMEM, could be indeed considered negligible, considering that neither pH variation nor effect on cell vitality could be detected. In addition to lanthionine, treatments were performed by supplying cells with phosphate (NaH_2_PO_4_, Thermo Fisher Scientific, Honeywell Fluka, 7558-80-7, Göteborg, Sweden) and calcium (CaCl_2_, Serva, 10043524, Heidelberg, Germany) to the final concentrations of 2.8 mM and 3 mM, respectively. The control cultures did not receive lanthionine treatment but were grown in normal medium (as an untreated control) or in medium with NaH_2_PO_4_ and CaCl_2_.

### 2.3. Measurement of the Intracellular Ca^2+^

The measurement of Ca^2+^ in the EA.hy926 cells was performed using a fluorescent Fura 2-AM probe (F1201, ThermoFisher Scientific, Milan, Italy). The reactions were performed according to the supplier’s protocol. Briefly, the DMEM medium was discarded, and the cells were washed twice in PBS (Dulbecco’s Phosphate Buffered Saline 1X, Microgem, Napoli, Italia) and incubated with 3 μM probe in PBS for 30 min at 37 °C in a humidified atmosphere with 5% CO_2_. At the end of incubation, the cells were washed with PBS. Then, fluorescence intensity was detected at λE_x_340 nm and λE_m_510 nm by a fluorescence multi-reader (Infinite 200, Tecan Trading AG, Männedorf, Switzerland) [35]. Experiments were performed in triplicate and the data were analyzed using GraphPad Prism software. Intracellular Ca^2+^ concentration was expressed as relative fluorescence intensity (RFI). RFI values were calculated by normalization to fluorescence intensity with respect to a control treated with DMEM, 2.8 mM CaCl_2_ and 3 mM of NaH_2_PO_4_, which was considered equal to 1.

### 2.4. RNA Extraction

The mRNA was extracted from cells using a mirVana™ PARIS™ kit (AM1556, ThermoFisher Scientific, Milan, Italy) according to the supplier’s protocol. The RNA concentration was measured by means of NanoDrop UV/Vis microspectrophotometry (ND-1000; NanoDrop Technologies, Wilmington, DE, USA). The samples were stored at −80 °C.

### 2.5. Reverse Transcription, PCR and qPCR

The cDNA was synthesized from 1 μg of total RNA. For reverse transcription, the QuantiTect^®^ reverse transcription kit 5× All in One RT Mastermix (G486, ABM, Richmond, BC, Canada) was used according to the supplier’s protocol. Reactions were performed in a Veriti^®^ 96-Well Thermal Cycler (Applied Biosystems, Foster City, CA, USA). The cDNA concentration was measured by means of NanoDrop UV/Vis micro-spectrophotometry. The cDNA samples were stored at −20 °C.

The qPCR experiments were performed using 200 ng od cDNA, a SsoAdvanced Univ SYBR Green Supermix (1725271, BioRad, Hercules, CA, USA) and a CFX96 Touch Real-Time PCR Detection System (BioRad, CA, USA). Target-specific PCR primers DKK1, ALPL, ANKH and ACTIN were obtained from BioRad (CA, USA) while RUNX2 and BMP2 were obtained from SIGMA (Sigma, St. Louis, MO, USA).

The amplification conditions were the following: 95 °C for 5 min; followed by 40 cycles of 94 °C for 5 s; the annealing/extension step was carried out at 60 °C for 30 s. The ΔΔCt method was used to analyze the relative changes in mRNA expression, with beta-actin as the normalization gene. Experiments were performed in triplicate and calculations were manually verified (“Users bulletin”, ABI PRISM 7700 Sequence Detection System 1997).

### 2.6. Protein Extraction

The proteins were extracted using an RIPA buffer (TCL131, HiMedia, Mumbai, India) containing a cocktail of protease inhibitors (cat.n°11836153001, Roche, Milan, Italy) and phosphatase inhibitors (4906845001, Sigma, St. Louis, MO, USA). Protein concentrations were determined according to Bradford (Bradford Protein Assay Kit; 5000001, BioRad, Hercules, CA, USA). The samples were stored at −20 °C in preparation for Western Blot analysis.

### 2.7. Western Blotting Analysis

The proteins were electrophoretically separated on precast gels 8–16% (456-1104, mini-PROTEANS^®^ TGX™ Gels, Bio-Rad, Hercules, CA, USA) and transferred onto 0.2 μm nitrocellulose membrane (1704158, Trans-Blot^®^ Turbo™, Bio-Rad, Hercules, CA, USA). Protein detection was performed using the following primary antibodies: anti-ERK (4595, Cell Signaling Technology, Danvers, MA, USA), anti-pERK (4370, Cell Signaling Technology, Danvers, MA, USA), anti-DKK1 (sc-374574, Santa Cruz Biotechnology, Inc., Dallas, TX, USA) and anti-GAPDH (5174, Cell Signaling Technology, Danvers, MA, USA) or anti-Tubulin (67305 Elabscience, Houston, TX, USA) were employed as loading controls as appropriate. The secondary antibodies anti-rabbit (NC27606, Immunoreagents Inc., Raleigh, NC, USA) and anti-mouse (Immunoreagents Inc., Raleigh, NC, USA) conjugated with horseradish peroxidase were utilized. The detection of the immunocomplexes was obtained by chemiluminescence, utilizing an Immobilon Western Chemiluminescent HRP Substrate (WBKLS0500, Millipore Corporation, Billerica, MA, USA) and a transilluminator (ChemiDoc™, Bio-Rad, CA, USA). The signal intensity was quantified with Image Lab software. All of the experiments were performed in triplicate.

### 2.8. Alizarin Red S Staining

The deposition of the calcified matrix was determined by Alizarin Red staining at 24 h, 48 h, 96 h and day 10. The cultured cells grown in 6-well plates (353046, Falcon, Glendale, AZ, USA) were fixed in 4% formaldehyde in phosphate-buffered saline (PBS) (TCL119, Himedia, Mumbai, India) for 15 min at 4 °C. The samples were then washed in distilled water and exposed to 2% Alizarin Red S pH 4.2 for 10 min (A5533, Sigma, St. Louis, MO, USA). After washing with distilled water, cells were observed by LEICA DM-IRB photomicroscopy using a 1×/5× magnification. Imaging was acquired by Optika camera software. Positive staining is represented as a red/purple color.

The stained cells were bleached with 10% (*w*/*v*) cetylpyridinium chloride (C0732, Sigma, St. Louis, MO, USA) in sterile water for 15 min at room temperature and the deposited calcified matrix was quantified by absorption at 562 nm by a multi-reader (Infinite 200, Tecan Trading AG, Männedorf, Switzerland). The results were expressed as arbitrary units (a.u). The experiments were performed in triplicate and the data were analyzed using the GraphPad Prism software.

### 2.9. Alkaline Phosphatase Activity Assay

The enzymatic activity of alkaline phosphatase (ALP) was assessed at 24, 48, 96 h, and on day 10 of the cell lysates. Cells were grown in Petri dishes until they reached 80% of confluence. After collecting the cells, the lysis buffer (20 mM Tris-HCl (77-86-1, Sigma, St. Louis, MO, USA); 0.5 mM NaCl (8521, Sigma, St. Louis, MO, USA); 0.25% Triton X-100 (8787, Sigma, St. Louis, MO, USA); 0.5 mM Phenylmethanesulfonylfluoride (RM1592; Himedia, Mumbai, India); 0.5 mM DL-Dithiothreitol (10197777001, Sigma, St. Louis, MO, USA); pH 7.4)) was added for 30 min incubation time on ice and then the cell lysates were centrifuged at 13,000 xg for 5 min and used for an ALP assay. The protein concentrations were determined according to Bradford (Bradford Protein Assay Kit; 5000001, BioRad, Hercules, CA, USA).

The alkaline phosphatase activity was determined on the supernatant by measuring the release of p-nitrophenol from disodium p-nitrophenyl phosphate. The reaction mixture contained disodium p-nitrophenyl phosphate, 0.5 mM MgCl_2_, 0.1 M diethanolamine phosphate buffer pH 10.5 (ready to use solution, N7653, Sigma, St. Louis, MO, USA) was mixed in a 1:1 volume ratio with the supernatant for 30 min at 37 °C. The reaction was stopped by adding an equal volume of 0.5 M NaOH. P-nitrophenol levels were measured by a multi-reader (Infinite 200, Tecan Trading AG, Männedorf, Switzerland) at 405 nm. One unit is defined as the amount of enzyme that hydrolyzes 1 nmol of p-nitrophenyl phosphate × min^−1^. Specific activity was expressed as nmol × min^−1^ × μg protein^−1^ and was corrected for the protein concentration of the cell lysates. All of the experiments were performed in triplicate and the data were analyzed using the GraphPad Prism software.

### 2.10. Statistical Analysis

The data are expressed as the means ± standard deviation (SD), except where otherwise specified. The results were analyzed using the statistics software GraphPad Prism Version 5.0 (GraphPad Software, San Diego, CA, USA). Statistical significance was determined as * *p* ≤ 0.05, ** *p* ≤ 0.01 and *** *p* ≤ 0.001 using one-way ANOVA test.

## 3. Results

### 3.1. Lanthionine Increases Intracellular Calcium Levels

Ca^2+^ is important for different processes such as muscle contraction and neuronal signaling [36,37]. Intracellular Ca^2+^ homeostasis can be disrupted by various stimuli, such as an excess due to Ca^2+^ uptake or the release of Ca^2+^ from cellular reservoirs. Studies demonstrated that treatment with CaxP induced an intracellular Ca^2+^ burst, promoting alterations in the cellular environment and cell death [38].

**Figure 2:**
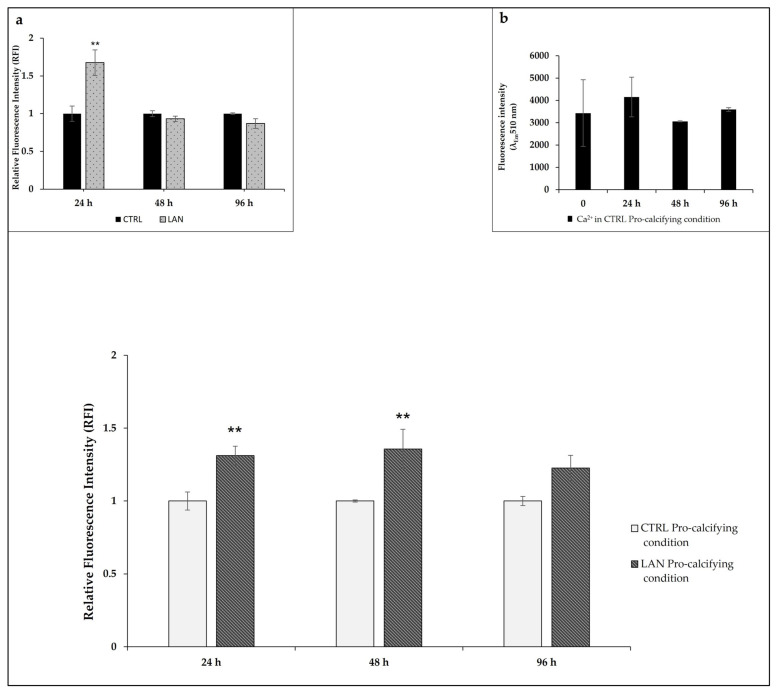
The effects of lanthionine on intracellular calcium levels using a fluorescent Fura 2-AM probe in EA.hy926 cultures. The effect of 1 µM of lanthionine treatment with 2.8 mM of CaCl_2_ and 3 mM of NaH_2_PO_4_ after 24 h, 48 h and 96 h on intracellular Ca^2+^ levels in endothelial cells. Ca^2+^ levels were detected using the fluorescent probe Fura 2-AM and were expressed as RFI as explained under “Materials and Methods.” Inset (**a**): the effect of 1 µM of lanthionine treatment after 24 h, 48 h and 96 h on intracellular Ca^2+^ levels in endothelial cells. Ca^2+^ levels were detected using the fluorescent probe Fura 2-AM and were expressed as RFI, as above. Inset (**b**): the effect of pro-calcifying conditions on intracellular Ca^2+^ levels after 0, 24 h, 48 h and 96 h of treatment. The columns represent the mean, while the error bars are the SD from three independent experiments; *p*-value versus untreated or treated control = ** *p* < 0.01, (according to the Anova test). CTRL, untreated control; LAN, lanthionine; CTRL Pro-calcifying condition, with 2.8 mM CaCl_2_ and 3 mM of NaH_2_PO_4_; LAN Pro-calcifying condition, lanthionine with 2.8 mM CaCl_2_ and 3 mM of NaH_2_PO_4_.

Calcium levels were measured by using a specific fluorescent probe (FURA 2-AM probe, 3 μM) after 24 h, 48 h and 96 h of treatment (Figure 2). Treatment with lanthionine alone provided overlapping results compared to our previous work [35]. In particular, in EA.hy926 cells, an increase in intracellular calcium content could be detected within 24 h, which returned to values similar to the untreated control at 48 h (Figure 2a) [35]. When considering the addition of lanthionine to the pro-calcifying medium (DMEM with 3 mM NaH_2_PO_4_ and 2.8 mM CaCl_2_), we detected a significant rise in intracellular calcium at 24 h and 48 h. No statistically significant time-dependent effects on intracellular Ca^2+^ levels could be observed as the result of cell incubation under pro-calcifying conditions (Figure 2b).

Intracellular Ca^2+^ levels were normalized with respect to the control sample (DMEM with 2.8 mM CaCl_2_ and 3 mM of NaH_2_PO_4_), as described under Section 2.

### 3.2. Changes in Gene Expression Involved in the Earliest Phase of Mineralizing Process

The calcification process is highly dependent on the expression of specific genes and on the biosynthesis of protein products, as well as on some post-biosynthetic regulation mechanisms. The principal genes correlated to the early stage of endothelium mineralization, as reported in Figure 1, were analyzed using Real Time PCR in EA.hy926 cells.

**Figure 3:**
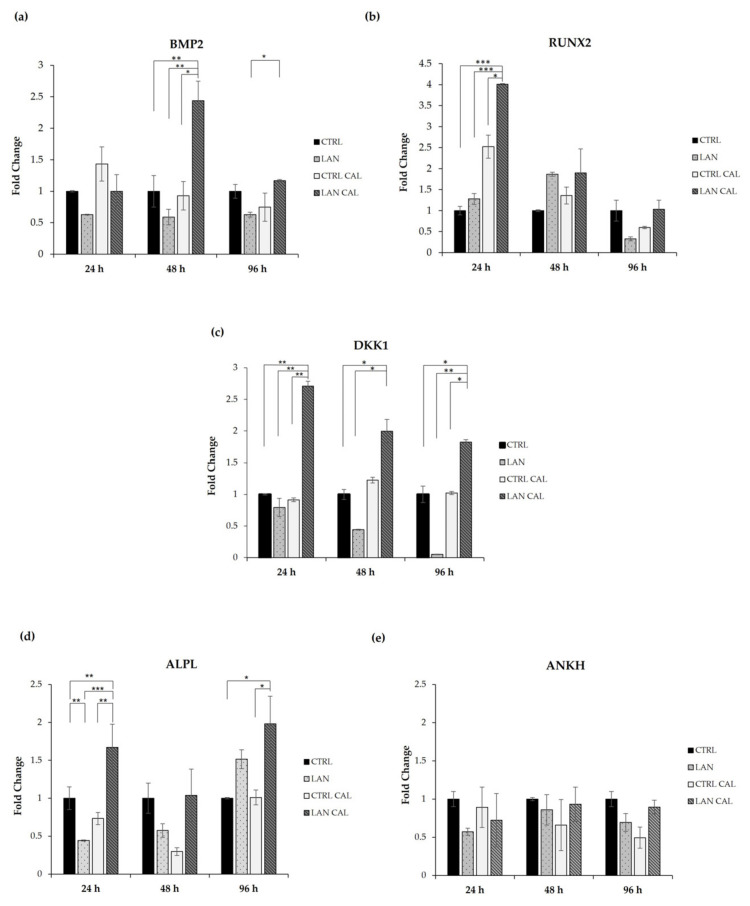
The effect of lanthionine on the expression of genes involved in the earliest phase of the mineralization process in EA.hy926 cell cultures. The relative expression levels of the genes, (**a**) *BMP2*; (**b**) *RUNX2*; (**c**) *DKK1*; (**d**) *ALPL*; (**e**) *ANKH*, using qPCR after 24 h, 48 h and 96 h of treatment, in comparison to the untreated control, are shown as a bar equal to 1: The columns represent the mean and error bars indicating the SD from three independent experiments; *p*-value versus the untreated control = * *p* < 0.05, ** *p* < 0.01; *** *p* < 0.001 (according to the Anova test). *BMP2*, Bone morphogenetic protein 2; *RUNX2*, Runt-related transcription factor 2; *DKK1*, Dickkopf WNT Signaling Pathway Inhibitor 1; *ANKH*, Inorganic Pyrophosphate Transport Regulator; *ALPL*, tissue-nonspecific alkaline phosphatase; CTRL, untreated control; LAN, lanthionine; CTRL CAL with 2.8 mM of CaCl_2_ and 3 mM of NaH_2_PO_4_; LAN CAL, lanthionine with 2.8 mM of CaCl_2_ and 3 mM of NaH_2_PO_4_.

Our results showed that treatment with lanthionine alone did not significantly increase the expression of any of the investigated genes in EA.hy926 cultures. We analyzed the effect of cell treatment with lanthionine in pro-calcifying conditions (DMEM with 2.8 mM CaCl_2_ and 3 mM of NaH_2_PO_4_), also evaluating the changes occurring in the sample treated with DMEM with 2.8 mM of CaCl_2_ and 3 mM of NaH_2_PO_4_ (Figure 3). The expression of *BMP2* is significantly increased in EA.hy926 cultures only at 48 h of treatment with lanthionine in pro-calcifying conditions (Figure 3a). *RUNX2* increased when lanthionine was added to pro-calcifying conditions only at 24 h, while no significant changes were observed at 48 h and 96 h. However, lanthionine alone tends to increase *RUNX2* expression at 48 h, followed by a reduction at 96 h of treatment, although not significant with respect to the control (Figure 3b). The expression of *DKK1* resulted in a rapid initial increase at 24 h, with a subsequent decrease in the expression at 48 h and 96 h (Figure 3c), while no change was observed in the control treated with Ca^2+^/P only. Instead, lanthionine alone reduces *DKK1* expression above all at 48 h and 96 h. *ALPL* increased at 24 h and at 96 h of treatment in EA.hy926 with respect to the control treated with Ca^2+^/P only. In this case, lanthionine slightly increased the expression of *ALPL* at 96 h, although not significantly (Figure 3d). *ANKH* did not show any significant change in its expression during lanthionine treatment (Figure 3e).

### 3.3. Effects of Lanthionine Alone and under Pro-Calcific Microenvironment on pERK/ERK and DKK1 Expression in EA.hy926 Cells

**Figure 4:**
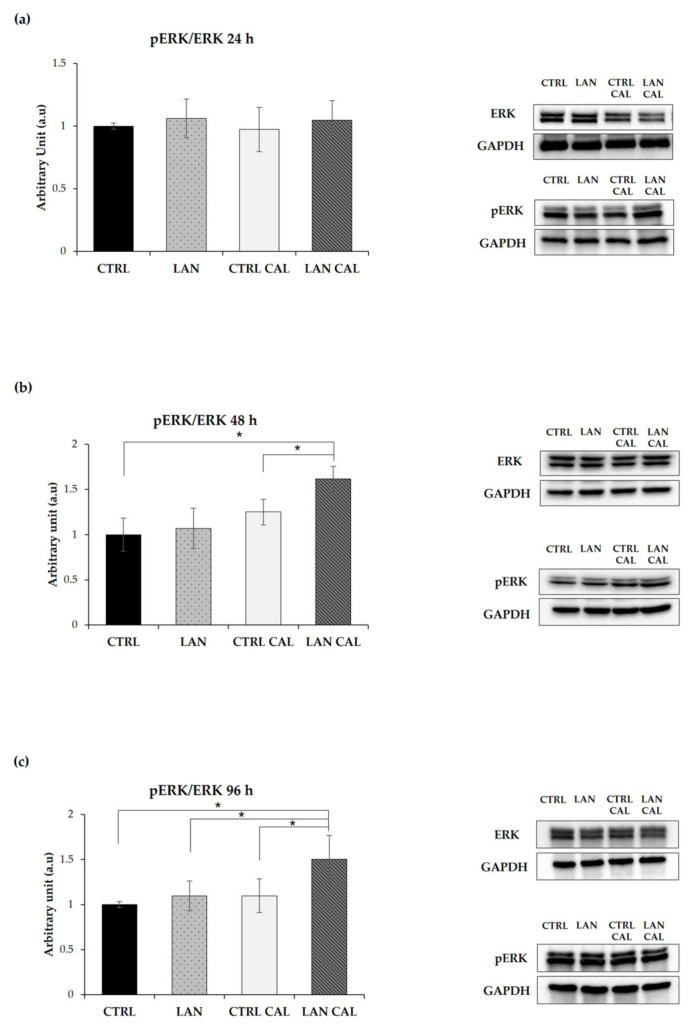
The effect of lanthionine alone and upon incubation with a pro-calcifying Ca*^2+/^*P medium on ERK phosphorylation in EA.hy926. The molecular characterization of intracellular ERK and pERK levels are expressed as a pERK/ERK ratio in endothelial cells after 1 µM of lanthionine and 1 µM of lanthionine with 2.8 mM of CaCl_2_ and 3 mM of NaH_2_PO_4_ treatment at 24 h, 48 h and 96 h. (**a**–**c**) Western blot analysis of pERK/ERK in endothelial cells; GAPDH is the loading control; the relative differences in protein levels shown (right of the panel) were quantitated by using Image Lab Software, normalized to GAPDH levels and expressed as a pERK/ERK ratio as arbitrary units (a.u). The untreated control is shown as a bar equal to 1. The columns represent the mean and error bars indicating the SD from three independent experiments; *p*-value versus untreated control = * *p* < 0.05, (according to Anova test). CTRL, untreated control; LAN, lanthionine; CTRL CAL, with 2.8 mM of CaCl_2_ and 3 mM of NaH_2_PO_4_; LAN CAL, lanthionine with 2.8 mM CaCl_2_ and 3 mM of NaH_2_PO_4_.

ERK1/2 are enzymes involved in various physiological cell processes and are crucially subject to the post-biosynthetic phosphorylation-dependent regulation of their activity [39]. We checked the effects of lanthionine on phosphoERK1/2, and no significant change was detected upon treatment with this amino acid with respect to the sample treated with Ca^2+^/P alone at 24 h of treatment (Figure 4a). However, phoshoERK1/2 increased significantly in EA.hy926 at 48 h and 96 h (Figure 4b,c), after treatment with lanthionine in a pro-calcifying incubation condition and with respect to the expression of the control treated with pro-calcifying conditions.

**Figure 5:**
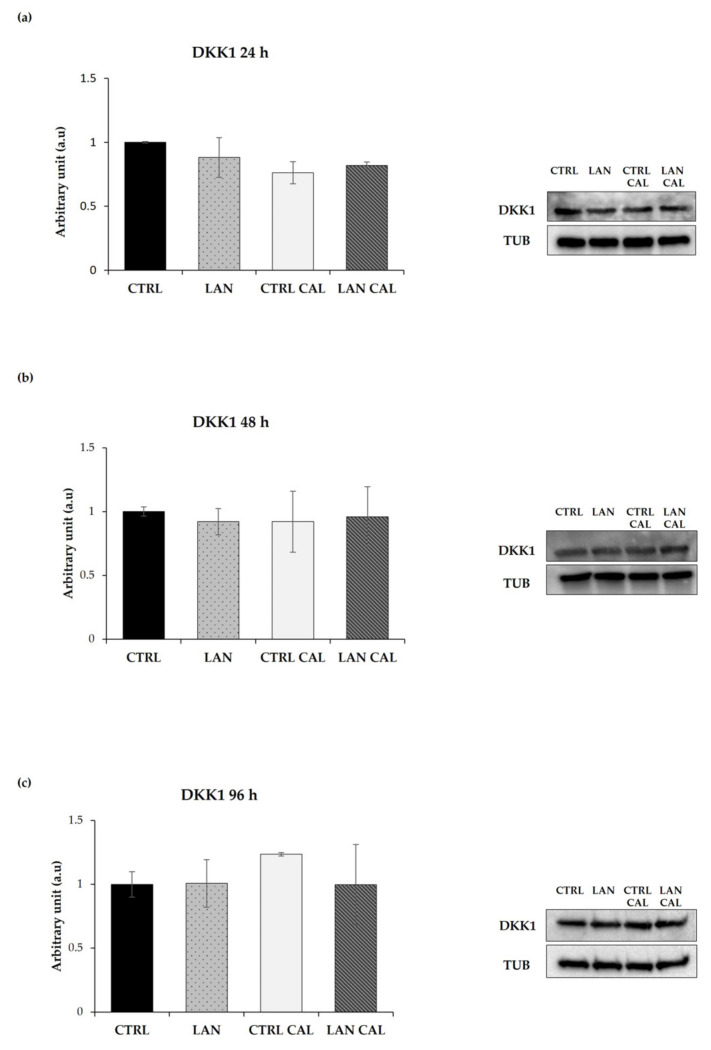
The Western Blotting analysis of DKK1 in EA.hy926 treated with lanthionine alone and with pro-calcifying Ca^2+/^P medium. The molecular characterization of intracellular DKK1 levels expressed in endothelial cells after 1 µM of lanthionine and 1 µM of lanthionine with 2.8 mM of CaCl_2_ and 3 mM of NaH_2_PO_4_ treatment at 24 h, 48 h and 96 h. (**a**–**c**) The Western Blot analysis of DKK1 protein abundance in endothelial cells; Tubulin is the loading control; the relative differences in protein levels shown (right of the panel) were quantitated by using Image Lab Software, normalized to Tubulin levels and expressed as arbitrary units (a.u). The untreated control is shown as a bar equal to 1. The columns represent the mean and error bars indicating the SD from three independent experiments, (according to Anova test). CTRL, untreated control; LAN, lanthionine; CTRL CAL, with 2.8 mM of CaCl_2_ and 3 mM of NaH_2_PO_4_; LAN CAL, lanthionine with 2.8 mM of CaCl_2_ and 3 mM of NaH_2_PO_4_.

DKK1 was the first discovered protein involved in antagonizing WNT/β-catenin [40,41,42]. Clinical studies have established the importance of DKK1 in the development of cardiovascular disease, showing a correlation between its high serum levels and the risk of cardiovascular disease [43]. We analyzed the levels of DKK1 in response to lanthionine in EA.hy926 cells during exposure to a pro-calcifying medium. The results are overall shown in Figure 5.

In contrast with the results from RT-PCR, cell incubation with pro-calcifying culture medium plus treatment with lanthionine exerted only a slight effect on the levels of DKK1 protein content at 24 h (Figure 5a), not showing any statistically significant change of its expression during lanthionine treatment, with respect to the untreated control at 48 h and 96 h (Figure 5b,c).

### 3.4. Lanthionine Effects on Calcium Deposition in EA.hy926 Cell

**Figure 6:**
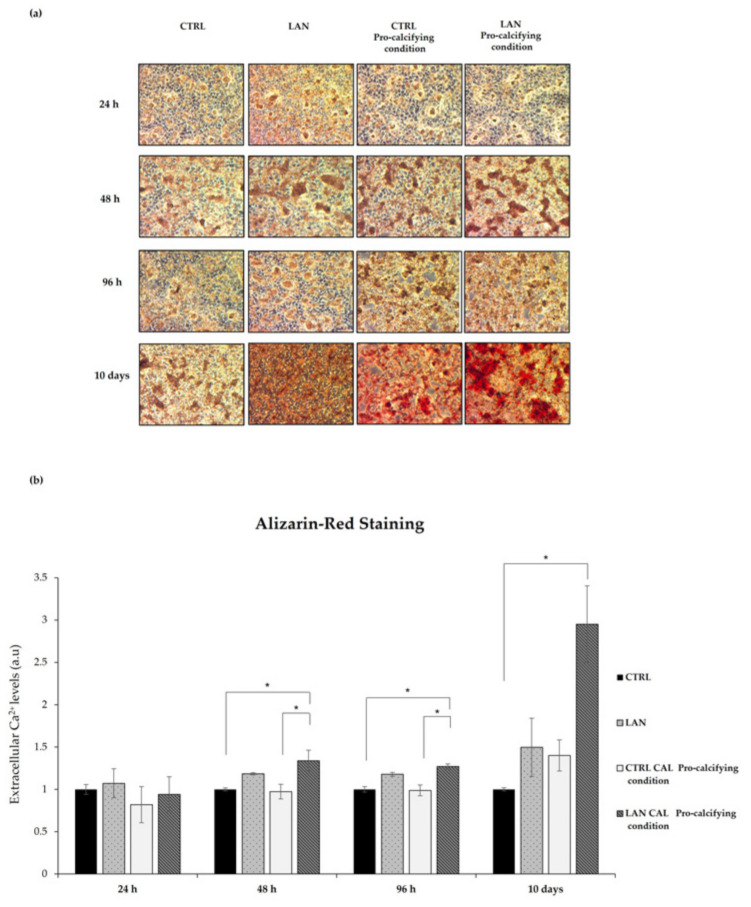
Lanthionine’s effects on calcium deposition in EA.hy926 cell cultures and medium incubated in pro-calcifying conditions. The Alizarin Red S staining and extracellular calcium content assay after 1 µM of lanthionine and 1 µM of lanthionine with 2.8 mM of CaCl_2_ and 3 mM of NaH_2_PO_4_ treatment at 24 h, 48 h, 96 h and 10 days. To understand the effects of lanthionine in a pro-calcifying condition, a control with 2.8 mM of CaCl_2_ and 3 mM of NaH2PO_4_ was used. (**a**) The images were observed by a LEICA DM-IRB microscopy equipped with a 5× objective Optika camera software; (**b**) the extracellular calcium levels were quantified using cetylpyridinium chloride at 562 nm (see “Section 2”) and normalized to protein content. The columns represent the mean and error bars indicating the SD from three independent experiments. The untreated control is shown as a bar made equal to 1. *p*-values versus untreated control = * *p* < 0.05, (according to Anova-test). CTRL, untreated control; LAN, lanthionine; CTRL Pro-calcifying condition, with 2.8 mM of CaCl_2_ and 3 mM of NaH_2_PO_4_; LAN Pro-calcifying condition, lanthionine with 2.8 mM of CaCl_2_ and 3 mM of NaH_2_PO_4_.

We adopted our EA.hy926 cell culture model in order to assess the potential effects of lanthionine on tissue mineralization. To do so, calcium deposition was monitored in EA.hy926 treated with lanthionine alone or in the presence of pro-calcifying Ca^2+^/P conditions. To understand if a prolonged treatment might be able to influence the extent of calcium deposits, we also performed a 10-day-long treatment. Alizarin Red S staining and extracellular calcium deposition assays were performed as described under Section 2. The results are shown in Figure 6a,b.

As shown in Figure 6a, treatment with lanthionine alone already caused an early increase in calcium deposition in the extracellular compartment, detected as red deposits, even at the shortest incubation time (24 h) (Figure 6a). When lanthionine treatment was accomplished in the presence of a pro-calcifying medium, at a 48 h, 96 h or longer incubation time, calcium deposition evidently reached the maximum in terms of color intensity (Figure 6a).

Calcium deposition, judged from Alizarin Red staining, at different times, was quantified using cetylpyridinium chloride, evaluating the absorbance at 562 nm. Figure 6b showed an increase in calcium content in a time-dependent manner, which substantially mirrored the result obtained using Alizarin Staining.

In conclusion, the Alizarin Red S staining and extracellular calcium content assays revealed that treatments with lanthionine and calcifying medium were able to greatly increase extracellular calcium content (Figure 6a,b) with a timing pattern which suggests that lanthionine may play an enhancing role in calcium deposition.

### 3.5. Alkaline Phosphatase Assay

**Figure 7:**
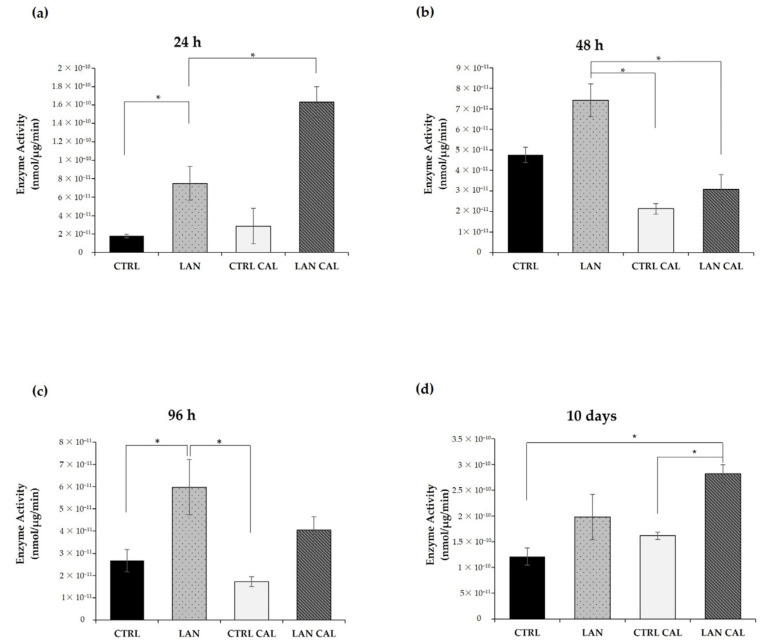
The alkaline phosphatase enzyme activity assay in Ea.hy926 cells upon lanthionine treatment in the presence of pro-calcifying medium conditions. The alkaline phosphatase enzyme assay after 1 µM of lanthionine and 1 µM of lanthionine with 2.8 mM of CaCl_2_ and 3 mM of NaH_2_PO_4_ treatment in 24 h (**a**), 48 h (**b**), 96 h (**c**) and 10 days (**d**). To understand the effects of lanthionine under pro-calcifying conditions, a control with 2.8 mM of CaCl_2_ and 3 mM of NaH_2_PO_4_ was tested. Enzyme activity corresponds to the release of p-nitrophenol from disodium p-nitrophenyl phosphate measured at 405 nm. One unit of enzyme is defined as the amount of enzyme that hydrolyzes 1 nmol of p-nitrophenyl phosphate x min^−1^. Specific activity is expressed as nmol × min^−1^ × μg and is corrected for protein concentration of the cell lysates. The columns represent the mean and error bars indicating the SD from three independent experiments; *p*-value versus untreated control = * *p* < 0.05, (according to Anova-test). CTRL, untreated control; LAN, lanthionine; CTRL CAL, with 2.8 mM of CaCl_2_ and 3 mM of NaH_2_PO_4_; LAN CAL, lanthionine with 2.8 mM of CaCl_2_ and 3 mM of NaH_2_PO_4_.

The specific activity of alkaline phosphatase (ALPL), an early indicator of calcification induction [44], was determined at 24 h, 48 h and 96 h in EA.hy926, upon treatment with lanthionine alone and under pro-calcifying conditions. Enzyme activity was higher with lanthionine alone or in combination with calcifying medium when compared with controls. As shown in Figure 7, treatment with lanthionine alone was associated with a biphasic increase in ALPL enzyme activity at 24 h and 96 h (Figure 7a,c). Treatment with lanthionine in a pro-calcifying medium induced an early and rapid increase of ALPL activity at 24 h (Figure 7a), followed by reduction at 48 h (Figure 7b) up to a strong increase detectable at 10 days (Figure 7d). Concerning the untreated control, the relevant variations detected were comparable among sample groups at the earlier time points (Figure 7a–c). Only in the last panel (Figure 7d), the ALPL activity in the control was increased compared to the previous sets of experiments. We referred this difference to the fact that the last set (Figure 7d) was obtained after a much longer incubation time (10 days).

## 4. Discussion

Ectopic calcification is a degenerative process, slow and silent, that often becomes clinically evident too late, when occlusive damage has occurred in a blood vessel. VC is often observed in CKD where it substantially contributes to the high cardiovascular risk detected in these patients when compared with otherwise healthy individuals [45,46].

CKD is also associated with benign nodular calcification as a result of the metastatic calcification that occurs when hydroxyapatite crystals, derived from abnormal serum calcium and phosphate, precipitate in normal tissues [47]. This condition is also associated with the presence of uremic toxins in CKD, which, in turn, create an altered micro-environment prone to entailing the pathophysiological alterations that lead to CV events [24]. In CKD, the altered environment induces the accumulation of modified amino acid residues in proteins, interfering with normal protein structure and activity [48,49].

In the current study, we demonstrated that the uremic toxin lanthionine, a non-proteinogenic amino acid, mainly generated as a side-product of the activities of transsulfuration enzymes in hydrogen sulfide (H_2_S) biosynthesis [50,51], is able to modify the endothelial homeostasis with the expression of specific markers involved in the mineralization process. The present results strengthen the notion that several metabolic derangements, which are operative in CKD and relevant to the calcification process, are indeed linked together. These include both sulfur amino acid and calcium and phosphate mineral alterations which, in the light of our results, appear pathophysiologically relevant. Our previous work demonstrated that lanthionine is able to increase intracellular Ca^2+^ levels [35] and promotes alterations in cardiac morphology and function in Zebrafish embryos [52].

Endothelial cells (ECs) constantly interact with blood components. In the context of CKD, dangerous signals from uremic toxins and cytokines may induce various molecular mechanisms, leading to injury. Studies demonstrated that uremic toxins stimulated ECs to produce extracellular vesicles (EV), promoting the calcium and phosphate effects of adjacent cells [53]. Dysfunction and injury of ECs were associated with various diseases, such as cardiovascular diseases (CVDs), cancer and kidney disease. To find possible targets in ECs to treat these conditions, different types of endothelial cells were used as a model. We employed immortalized Ea.hy926 cell cultures with similar characteristics with primary human endothelial cells, such as HAECs, that are more stable and easier to maintain than primary human cultured ECs [54]. In recent years, different studies investigated vascular calcification utilizing immortalized line, such as mouse VSMC line MOVAS-1 cells with a SMCs-specific phenotype [55,56,57]. Moreover, other studies evaluated aortic valve disease and its related processes, as well as calcification, using immortalized cell lines derived from sheep and rats [58].

In this study, we wanted to investigate the possible role played by lanthionine to promote changes in markers involved in the mineralization process. EA.hy926 cells were exposed, using calcium and phosphate, to pro-calcifying-damaging condition metabolic experimental conditions comparable to those present in CKD subjects. We investigated not only the effects of lanthionine, but also the potential interactions of this amino acid within an altered pro-calcifying medium that resembles the intrinsic uremic milieu conditions. In fact, the strictly interconnected dysregulation of Ca^2+^ and phosphate in renal disease plays a crucial role in the pathogenesis of calcification. The high prevalence of these alterations requires that control of mineral and uremic metabolic derangement is a therapeutic target to try to minimize disease progression and vessel damage. As reported in our previous work, after a short-term treatment (12 h), lanthionine alone was able to enhance calcium levels, which then decreased after 48 h of treatment [35]. In pro-calcifying conditions, this effect is maintained over time. The importance of intracellular calcium homeostasis has already been studied, showing that different stimuli could easily alter the cellular environment with the presence of a mineral nucleation site [59]. Consistently with this line of evidence, in our recent work, we demonstrated a correlation between the presence of lanthionine, the alteration of calcium homeostasis and inflammation in CKD patients [34]. In this work, our hypothesis was to help recognize the role of the endothelium as an early site of pathologic remodeling which contribute to vessel injury. In fact, it may be difficult to identify the relevant roles of the intima and media in the overall calcification-related events, since these tunicae participate in common pathophysiological mechanisms. Moreover, the endothelium influences angiogenesis, particularly in the areas of intima thickening and calcification [60,61].

From a molecular standpoint, we used Real Time PCR to analyze the expression of the earlier genes involved in the mineralization process. Bone morphogenetic protein 2 (*BMP2*) is a regulator of tissue homeostasis and bone formation, but *BMP2* in endothelial cells is also involved in vascular inflammation and angiogenesis which are strongly associated with the calcification processes [62,63]. Treatment with lanthionine and pro-calcifying conditions induces an increase of *BMP2* within 48 h. As reported in other works, the increase of *BMP2* is necessary for the expression of the critical transcription factors involved in the mineralization, such as Runt-related transcription factor 2 (*RUNX2*) [64].

*RUNX2* is an early mineralization marker, and a major contributor of its increase was given by the presence of inorganic phosphate and by the reactive oxygen species (ROS) produced in response to high phosphate [31] (Figure 1). *RUNX2* acts as stimulator of angiogenesis and as a transcription factor that increases the expression of other genes, such as tissue-nonspecific alkaline phosphatase (*ALPL*) [28,65]. We hypothesize that lanthionine, while enhancing the local effects of Ca^2+^ content, produces a cellular environment that promotes the synergism between CaxP with the expression of *RUNX2* at 24 h sensitive to Ca^2+^ content, followed by the increase of *BMP2* after 48 h of treatment (Figure 3a,b).

The levels of phosphate and Ca^2+^ also influence the expression of *ALPL* and Inorganic Pyrophosphate Transport Regulator (*ANKH*) that are involved in pyrophosphate metabolism with opposite roles [66]. The *ALPL* gene encodes for a membrane-bound glycoprotein hydrolase that removes phosphate groups from different molecules or increases the hydrolysis of pyrophosphate, an inhibitor of hydroxyapatite formation. In humans, there are three tissue-specific ALPL isoenzymes, placental (PLAP), Germ Cell (GCAP) and Intestinal (IAP), which are abundant in mineralizing tissues, kidneys and in the central nervous system [29]. Studies demonstrated that the overexpression of this enzyme promotes the osteogenic potential of ECs [67]. We hereby observed an increase in *ALPL* expression after 24 h of treatment, when the level of intracellular Ca^2+^ increases, followed by a reduction.

The protein encoded by *ANKH* gene regulates PPi levels through the release of PPi in the extracellular environment [34]. *ANKH* exerted a protective effect with a negative correlation with the mineralizing process. In fact, the expression of *ANKH* was found to be decreased in human artery walls in patients with CKD or atherosclerosis [68]. This agrees with our results, where the levels of expression of *ANKH* are equal to those detected in the control. *DKK1* encodes a secreted protein characterized by two cysteine-rich domains that bind to the LRP6 co-receptor and inhibits β-catenin-dependent Wnt signaling [30]. Our results showed that *DKK1* expression peaked at 24 h of treatment, then decreased during the following time course.

We hypothesized that the mineralization process, in our experimental model, could be triggered within 24 h from the beginning of treatment. This interpretation is supported by the increase in early osteogenic markers such as *RUNX2*, *DKK1* and *ALPL.* Hortells et al. found that DKK1 expression overlapped with that of ALPL, hypothesizing a possible role of DKK1 in the modulation of ALPL expression [30]. In our experimental conditions, the increase in the expression of these two genes occurred within 24 h, concurrently with the intracellular calcium increase. However, we could not detect any increase in the DKK1 protein product, possibly indicating that other posttranslational mechanisms may be operative [69,70]. Independent studies have also demonstrated that calcium and phosphate regulated the activation of ERK1/2 [71,72,73]. It has been also demonstrated that ERK1/2 is involved in the phosphorylation of *RUNX2*, enhancing its transcriptional activity [33,74].

Moreover, ERK1/2 was able to promote a BMP2-induced pro-inflammatory phenotype in endothelial cells [75]. Our results demonstrate an activation of ERK1/2, particularly after 48 h of treatment with lanthionine in pro-calcific condition, a time course that overlaps with the expression of *BMP2,* as shown in Figure 3a.

Small calcium deposits are the leading actor of the early phase of calcification until the formation of massive mineral deposits takes place. We have shown that extracellular calcium deposits started to increase after 48 h of treatment with lanthionine in a pro-calcifying condition, an effect most visible after 10 days. It is also notable that lanthionine alone may induce microscopic calcium deposits and an increase in the calcium content after 10 days (Figure 6). This result may suggest that lanthionine alone could be also able to induce ectopic mineralization, since it needs time to activate the various molecular processes involved. In fact, when lanthionine is present under pro-calcifying conditions, it could upgrade the synergism between CaxP, which may activate more rapidly and affect the mineralizing pathological processes. Another factor that promotes mineralization is the increase in ALPL enzyme activity [76,77,78]. The data obtained may indicate, again, that lanthionine, in pro-calcifying conditions, promotes the mineralizing process, since a pro-calcifying treatment in the absence of lanthionine did not produce substantial ALPL enzyme alterations.

## 5. Conclusions

The present results support the notion that, under predisposing metabolic mineral disorder conditions, lanthionine may act synergistically with Ca^2+^/P ions. In these conditions, lanthionine may contribute to uremic toxicity in CKD by inducing the expression of specific molecular early markers involved in the mineralization process. The latter may indeed be considered as potential risk factors for “marker-guided” and “marker-targeted” therapeutic approaches in the future. One limitation of this study is the use of immortalized endothelial cells. Although their survival is dependent upon culture conditions, they do not fully develop their natural networks. Vasculature is a complex system of interactions among the endothelium and the other cells, tissues and circulating factors, although both primary cell cultures and in vivo animal models may be useful to elucidate the pathophysiology of calcification. Nevertheless, immortalized cell cultures have been proven to be quite suitable and easy to handle in the study of the mechanisms of uremic toxins, such as lanthionine, on endothelial-derived cell systems [35,54,79]. Our study strengthens the interpretation that mineralization in CKD is not simply the result of metastatic calcification deposition in otherwise normal vascular tissues as a result of the mineral and bone disorder associated with endocrine derangement. The overall conclusions, of present and previous observations, support the view that the association of local vascular inflammatory and dystrophic alterations may determine the ideal microenvironment for further damage. This, in turn, supports the hypothesis that the endothelium, together with vessel tunica media, participates in this pathophysiological process by playing a key role, as proven by the fact that several biomarkers of endothelium damage have been primarily found altered in uremia [80,81,82,83]. The discovery of early biomarkers is an excellent starting point for being able to identify factors that can prevent or delay disease progression.

## Data Availability

Not applicable.
